# Fabrication and characterization of solid lipid nano-formulation of astraxanthin against DMBA-induced breast cancer via Nrf-2-Keap1 and NF-kB and mTOR/Maf-1/PTEN pathway

**DOI:** 10.1080/10717544.2019.1667454

**Published:** 2019-09-26

**Authors:** Tao Sun, Jun Gao, Dan Han, Hongyan Shi, Xianqiang Liu

**Affiliations:** a Department of Thyroid Breast Surgery, Jinan Central Hospital Affiliated to Shandong University, Jinan, China;; b Eye, Plastic and Oral Wards, Jinan Central Hospital Affiliated to Shandong University, Jinan, China;; c Department of ENT (Ear–Nose–Throat), Jinan Central Hospital Affiliated to Shandong University, Jinan, China

**Keywords:** Astraxanthin, solid lipid nanoparticles, breast cancer, antioxidant, HMG-CoAR, NF-kB, metabolizing enzymes, Nrf-2, Keap1

## Abstract

In the current experimental study, we scrutinized the chemoprotective effect of astraxanthin against the 7,12-dimethylbenz(a)anthracene (DMBA)-induced breast cancer via Nrf-2-Keap1 and NF-kB and mTOR/Maf-1/PTEN pathway. The double emulsion solvent displacement method was used for the preparation of astraxanthin solid lipid nanoparticles (SLN). SLNs were appraised for entrapment, potential, size, drug-release performance, and gastric stability. DMBA (8 mg/kg) was used for the induction of breast cancer. Tumor weight, body weight, and tumor incidence were estimated at a regular interval. Different biochemical parameters such as Na+/K+, Ca2+, and Mg2+ activity, antioxidant, lipid, glycoprotein, phase I and II biotransformation enzymes, mitochondrial TCA cycle, and carbohydrate metabolizing enzymes were estimated. Keap1-Nrf-2, associated HO-1, and NF-kB expressions were estimated. Moreover, it estimated the mRNA expression of LXR (α,β), HMG-CoAR, PTEN, Maf1, PI3K, mTOR, Akt, FASN, and ACC1. AX-SLN reduced the tumor incidence, tumor weight, and increased the body weight. AX-SLN exhibited the protective effect against the LPO, enzymic (SOD, CuZnSOD, MnSOD, GPx, and CAT), and nonenzymic (GSH) in the serum, mammary gland, renal, and hepatic tissues. AX-SLN reduced the p-AKT which is accountable for the reduction in the NF-kB expression and also reduced the expression of Keap1 and NF-kB along with increasing the expression of HO-1 and Nrf-2. Further, AX-SLN significantly altered the mRNA of LXR (α,β), HMG-CoAR, PTEN, Maf1, PI3K, mTOR, Akt, FASN, and ACC1. On the basis of the results, we can conclude that AX-SLN inhibits the mammary gland carcinogenesis via Nrf-2-Keap1, NF-kB, and mTOR/Maf-1/PTEN pathway.

## Introduction

Among the common cancers, such as breast, lung, colorectal, and bronchus, breast cancer was diagnosed most commonly among women. Breast cancer is the most dreadful disease among all cancers and it mostly affects women among all other cancers (Eheman et al., 2012; Cronin et al., 2018). The incidence of breast cancer is very common among women and it is the second most common cause of cancer-related death. According to the previously published data, studies suggest that breast cancer alone accounts for 6.29% of all new cancer diagnosed in women. In the year 2015, 15.2 million cases were detected and 8.9 million of the affected patients died (Ferlay et al., 2010; Akram et al., 2017). The mortality and morbidity rate of breast cancer is highest throughout the world (Arbyn et al., 2011; Ferlay et al., 2014). The available treatment of cancer involves either suppression of exposure of an individual to a known carcinogen to extend possible and/or seeking advantage of the inhibitors of carcinogenesis for eventual application as anticancer agents (Ojeswi et al., 2012). The most common risk factors of breast cancer are age, estrogen exposure, mutation in tumor suppressor gene, obesity, and environmental pollutants (Rakha et al., 2007; Li, 2010). Moreover, breast cancer is also linked with estrogen exposure and age. Steroidal hormones such as estrogen can induce and expand breast cancer. Currently, only two selective estrogen receptor modulators (SERMs) such as raloxifene and tamoxifen were approved from the United States FDA for the treatment of breast cancer in women who are highly susceptible (Brody & Rudel, 2003; Millikan et al., 2008). But due to the serious side effects, these drugs have limitations. Moreover, the effects of these drugs may not completely eliminate the chance of induction of breast cancer; therefore, there is a scope for newer interventions (Sutradhar & Amin, 2014).

Breast cancer is categorized via cellular and oxidative imbalance due to the formation of tumors or lump in the tissue (Haldosén et al., 2014; Nwabo Kamdje et al., 2014). A previous research suggests that oxidative stress plays an important role in the carcinogenesis of mammary carcinoma (Karimi et al., 2019; Marcom, 2017). Research also suggests that oxidative imbalance may bring the physiological and structural changes to the normal cell biology which may further contribute to various deteriorative injuries and also leads to certain pathologies like cancer (Karimi et al., 2019; C.R. UK, 2014; Marcom, 2017). The multidysfunction brings mutagenic and genomic instability in various cell organelles like nucleus, endoplasmic reticulum, mitochondria, etc. and leads to the expansion of cancer. Various studies reported that ROS concentration was higher in the breast cancer cells and these free radical/ROS act as a promoter to cancer expansion, progression, and proliferation (Matés et al., 1999; Liou & Storz, 2010). Various clinical studies have been executed for effectively combating breast cancer (Klaunig et al., 2010). Available treatments have limitations due to their side effects and limited effects on tumors develop due to the therapeutic resistance and treatment-related morbidity (Halliwell, 2006; Valko et al., 2006). Therefore, there is an urgent need of newer drugs for the treatment of breast cancer with minimal side effects. The treatment should produce chemo-protective effects along with reduction in the illness and deaths related to the breast cancer.

7,12-Dimethylbenz(a)anthracene (DMBA) is the most dangerous polyaromatic hydrocarbon having the widest environmental distribution and it is also used for experiments (Anbuselvam et al., 2007; Davison et al., 2013). During the carcinogenic process, DMBA metabolizes and generates various reactive metabolic intermediates which further form the stable DNA adducts that are genotoxic and mutagenic initializing carcinogenesis, similar to MC in humans (Padmavathi et al., 2006; Davison et al., 2013). The oxidative misbalance regulates various protein and gene expressions which alter various signaling pathways and processes such as angiogenesis, apoptosis, cell growth, DNA repair, proliferation, invasion, etc (Vurusaner et al., 2012; Semenza, 2017).

Solid lipid nanoparticles (SLNs) are considered as the novel drug delivery system that is developed in the year 1991 and has attracted the drug delivery system due to its augmented oral bioavailability for poorly water-soluble drugs. SLNs were used as the alternative carrier system to the traditional colloidal carriers including liposomes, emulsions, and polymeric micro and nanoparticles. SLNs are considered as colloidal nano-structured carriers with particle size ranging between 1 and 1000 nm (Yadav et al., 2013; Naseri et al., 2015). SLNs are considered as the effective alternates over the polymeric nanoparticles due to controlled drug release, enhanced stability, low cost of excipients, high drug loading capacity, ease of preparation of hydrophobic/lipophilic drugs, and, finally, improved biopharmaceutical performance (Müller et al., 2002; Pandey et al., 2005; Yadav et al., 2013). Various researchers suggested that the SLNs up-regulated the oral bioavailability of drugs via transcellular and paracellular pathway (Pandey et al., 2005; Bunjes & Unruh, 2007). SLNs drug can be absorbed via the small intestinal epithelial cells after lipolysis (Müller et al., 2002; Das & Chaudhury, 2011). SLNs particularly contain the long-chain triglycerides, and also undergo drug absorption via intestinal lymphatic pathways (Müller et al., 2002; Mei et al., 2003). After oral administration of SLNs, they cannot be absorbed in their intact form in the gastrointestinal (GI) tract (Sarmento et al., 2007). During lipolysis, triglycerides are regularly digested into fatty acid and monoglycerides. Several factors such as empty stomach, gastric emptying rate, percentage of fat and oil, and presence of lipolysis inhibitor influenced the extent rate of lipolysis (Müller et al., 2002; Prow et al., 2011; Yadav et al., 2013; Naseri et al., 2015). Subsequently, various drugs that are nano-form encapsulated are directly transferred to the epithelial cells via the unstirred water layer during the lipolysis process and are then absorbed via intestinal epithelial cells in the form of mixed micelles of free molecules via passive diffusion, which further starts the up-regulation of oral bioavailability. Positively, SLNs also enhance the bioavailability of insoluble drugs via other routes, such as improving dissolution process of nano-aqueous drug for enhancing the solubility, as well as improved the distribution, transport, and absorption of the drug to their nanometer-scale particle size (Yadav et al., 2013; Naseri et al., 2015). Therefore, SLNs also have the advantages such as controlled and sustained drug release, nontoxic or no side effects, beneficial and targeted drug delivery system, effectual large scale production as well as enhancing the stability of unstable substances. It can be administrated via various routes such as oral, intravenous, topical, transocular, and transalveolar modes. Additionally, SLNs system can be lyophilized to ensure the long-term stability of the drug.

## Material and methods

### Chemicals

Breast cancer cells namely MCF-7 (hormone receptor-positive), MDA-MB-468 (triple-negative), and SK-BR-3 (HER2 positive) were purchased from the American Type Culture Collection. DMBA and astraxanthin were purchased from the Sigma Aldrich (St. Louis, MO). The pro-inflammatory cytokines kits such as IL-6, TNF-α, and IL-1β were purchased from the Sigma Aldrich (St. Louis, MO). All other chemicals used in the experimental study were of analytical grade.

### Solid lipid nanoparticles

For the preparation of solid lipid nanoparticles of ganoderic acid, the solvent displacement method was used with minor modification. Briefly, ganoderic acid was mixed in the organic phases before the incorporation of lecithin 90 and tristearin. The lecithin 90 and tristearin were taken in different ratios. Tween 20 was separately dissolved in the distilled water and mixed with the lipid. The solution was stirred at 3000 rpm for 2 h and purified to separate the SLNs.

### Zeta (f) potential, particle size (PS), and polydispersity index (PDI)

For the determination of PDI and PS, the dynamic light scattering (DLS) model was used. Briefly, the prepared nano-formulation was diluted 200 times using the aqueous phase followed by vigorous shaking to get 100–300 k counts/s. For the determination of Zeta (f) potential, Malvern instrument (Malvern Instruments Worcestershire, UK) was used.

### Scanning electron microscopy (TEM)

TEM spectroscopy was used for the microscopical evaluation of the prepared AX-SLN.

### Encapsulation efficiency and drug loading capacity

The purpose of the drug-loading capacity was to estimate the drug content present in the nanoparticles after separation into the medium. The entrapment capacity is used for the determination of drug content entrapped/adsorbed into the AX-SLN.

The loading capacity and the entrapment efficiency (EE) of AX-SLN were determined via separating the free astraxanthin from the SLN at 12 *g* rpm for 20 min. The following formula was used for the estimation of drug loading and entrapment efficiency capacity:

$$\mathrm{Drug}\;\mathrm{Loading}\;\mathrm{Capacity}=\frac{\mathrm{Entraped}\;\mathrm{Drug}}{\mathrm{Weight}\;\mathrm{of}\;\mathrm{Nanoparticles}}\;\times 100$$

$$\mathrm{Entrapment}\;\mathrm{Efficiency}\;\left(\%\right)=\frac{\mathrm{Total}\;\mathrm{Amount}\;\mathrm{of}\;\mathrm{Astraxanthin}-\mathrm{Amount}\;\mathrm{of}\;\mathrm{Astraxanthin}\;\mathrm{in}\;\mathrm{supernatant}}{\mathrm{Amount}\;\mathrm{of}\;\mathrm{Astraxanthin}}\;\times 100$$

### *In vitro* gastrointestinal stability

The *in vitro* gastrointestinal stability study was performed for the estimation of the stability of AX-SLN in the GI track. Briefly, the optimized AX-SLN was transferred into the simulated gastric fluid (200 mL) for 3 h and then to intestinal fluid for the next 9 h. At the time of 3 h, 1 ml of the sample was withdrawn and transferred to the cuvette for determination of particle size, entrapment efficiency, and zeta potential.

### *In vitro* drug release study

The *in vitro* drug release study was performed for the estimation of drug release pattern of AX-SLN using the dialysis method with minor modification. Briefly, AX-SLN was incubated in 50 ml of phosphate buffer saline (pH = 7.4) at 37 °C. After that, the aliquot samples were collected at various time intervals and the amount of astraxanthin was estimated for calculating the cumulative drug release at different time intervals.

### Animals

The current experimental study was performed according to the Animal Welfare Guidelines. In this study, the rats (female; weight 150–180 g; age 8–10 weeks) were kept in the standard laboratory condition (temperature 25 ± 2 °C; 12/12 h light and dark cycle). The rats were fed with the standard rat chow and water *ad libitum*.

### Experimental design

The rats were randomly divided into five groups and categorized as follows:

Group I: normal control
Group II: DMBA control
Group III: DMBA + astraxanthin (5 mg/kg)
Group IV: DMBA + astraxanthin (50 mg/kg)
Group V: DMBA + SLN-AX

The rats received DMBA (8 mg/kg, b.w.) intraperitoneally and after 1 week, they received the same drugs for 20 weeks as discussed in the above treatment. At the end of the experimental study, the rats were anesthetized and the blood samples were collected from rats of all the groups. The sample was centrifuged to separate the plasma for further estimation.

Lastly, the rats were sacrificed via cervical dislocation and different tissues were separated for further estimation.

### Estimation of body weight and tumor parameters

The bodyweight of all the rats was estimated at regular time intervals. The tumor volume of the experimental rats was estimated using the previous formula with minor modifications.

### Hepatic parameters

The hepatic parameters such as alanine aminotransferase (ALT), aspartate aminotransferase (AST), and alanine phosphatase (ALP) were estimated using the manufacturer’s instruction. Gamma-glutamyl transpeptidase (GGT) and lactate dehydrogenase were estimated using the previously reported method with minor modifications.

### Phase I and II enzymes

The phase I and II enzymes were estimated in the mammary gland tissue. The mammary gland tissue was separated in all groups of animals and washed with ice-cold sterile KCl, homogenized and centrifuged for separating the S9 fraction of microsomes for further analysis. Phase I enzymes such as cytochrome b5 and cytochrome P450 were estimated using the previous method with minor modifications. Phase II detoxification enzymes such as quinone reductase (QR) and glutathione S-transferase (GST) were estimated using the previously reported method with minor modifications.

### Antioxidant parameters

The antioxidant parameters such as superoxide dismutase (SOD), glutathione (GSH), catalase (CAT), glutathione peroxidase (GPx), vitamin (C and E), GST, GR, Cyp450, and Cyt-b5 were estimated using the previously reported method with minor modifications.

### Hepatic tissue injury markers

Hepatic tissue injury markers such as alpha-fetoprotein (AFP), aspartate transaminase (AST), alkaline phosphatase (ALP), alanine transaminase (ALT), and Gamma-Glutamyl Transferase (GGT) were estimated using the instruction provided by the manufacturer in the kits.

### Estimation of pro-inflammatory cytokines

Pro-inflammatory cytokines such as IL-6, IL-1β, and TNF-α were estimated using the instruction provided by the manufacturer of the ELISA kits.

### Estimation of HMG-CoAR

At the end of the experimental study, HMG-CoAR enzyme was estimated using the ELISA kits following the instructions provided by the manufacturer.

### Determination of LXR (α and β), HMG-CoAR, PI3K, mTOR, Akt, PTEN, and Maf1

At the end of the experimental study, the mammary tissue was washed with phosphate buffer saline (PBS) and trizol reagent was extracted with total RNA, stored at −80 °C for 2 h and then centrifuged (13 *g* rpm) at 4 °C for 15 min. cDNA (Bio-Rad RT-PCR kits, Bio-Rad, Hercules, CA) was synthesized with total RNA (1 μg) following the instructions provided by the manufacturer. The qPCR of all genes was performed using specific backward and forward primers presented in Supplementary Table 1.

### Data analysis

Results are expressed as mean ± SEM. The total variation present in a set of data was estimated by one-way analysis of variance (ANOVA) followed by Dunnet’s test. *p* < .05 was considered significant.

## Results

### Validation of optimized model

The prepared AX-SLN particles were found in spherical shape with uniform size distribution. The prepared formulation was optimized using different parameters such as the particle size with an average size of 172.5 nm, showing the validly of predicted model. The prepared AX-SLN showed the mean percentage, entrapment efficiency, and mean loading capacity as 60%. Transmission electron microscopy (TEM) showed the spherical shape and nano-size range of the prepared AX-SLN (Supplementary Figure 1).

### *In vitro* release of AX-SLN

The permeation studies of AX-SLN and control were evaluated during the *in vitro* permeation experiment. The skin permeation profiles of AX-SLN were standardized in accordance with the Fick’s diffusion law. Statistical data for the 24 h study showed a higher flux for AX-SLN as compared with that of the control (AX solution) (Supplementary Table 2), whereas the cumulative amount of AX permeated from SLN was 3.75 times higher than that of the control.

### Effect on tumor incidence

Supplementary Table 3 shows the effect of the AX-SLN on the tumor incidence of DMBA-induced breast cancer rats. The normal control group rats did not exhibit any sign and symptom of a tumor in the mammary gland. DMBA-induced rats showed 100% mammary gland tumor in the rats. DMBA-induced rats showed the pulmonary metastases (100%), lymph node metastases (100%), and adenocarcinoma (100%). On the one hand, DMBA-induced breast cancer rats treated with AX (5 mg/kg) demonstrated the pulmonary metastases (58.45%), lymph node metastases (50%), and adenocarcinoma (45.47%) and AX (50 mg/kg) demonstrated the pulmonary metastases (45.49%), lymph node metastases (37.54%), and adenocarcinoma (37.54%). On the other hand, AX-SLN demonstrated the pulmonary metastases (25%), lymph node metastases (12.5%), and adenocarcinoma (12.5%).

Supplementary Table 4 shows the number of rats having a tumor. The normal control group rats did not show any type of tumor formation. DMBA-induced rats exhibited the tumor incidence of 100% and tumor burden of 95 g. DMBA-induced rats treated with the AX exhibited the tumor incidence (58.45 and 45.49%) and tumor burden (72.5 and 45.62 g) at a dose level of 5 mg/kg and 50 mg/kg, respectively.

### Effect on body weight

**Figure 1.**
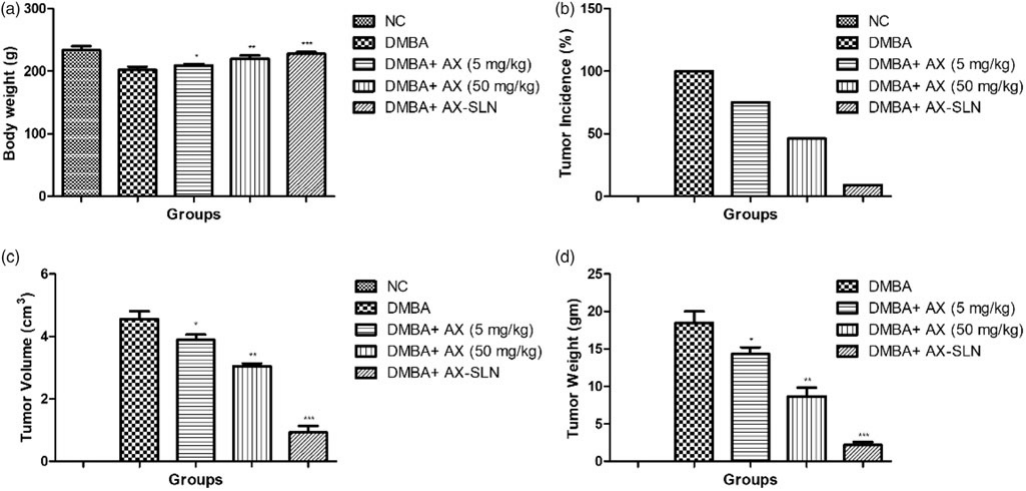
The effect of AX-SLN on body weight and tumor incidence of DMBA-induced breast cancer rats. (a) Bodyweight; (b) tumor incidence, (c) tumor volume, and (d) tumor weight. Each value shows the mean ± SEM, where the treated group rats were compared with the DMBA-induced group rats. Statistical analysis was done by one-way ANOVA followed by Dunnett’s multiple comparison. *p < .05, **p < .01, and ***p < .001.

Figure 1(a) illustrates the effect of AX and AX-SLN on the DMBA-induced breast cancer rats. The normal control group rats showed increased body weight throughout the whole experimental study. DMBA-induced group rats showed reduced body weight as compared to the normal control and other treated group rats. The AX (5 mg/kg) group rats showed increased body weight as compared to the DMBA-induced breast cancer group rats. A similar result was observed in the AX (50 mg/kg)-treated group rats. The AX-SLN-treated group rats showed increased body weight almost near to the normal control group rats.

Figure 1(b) exhibits the effect of AX on the tumor incidence of DMBA-induced breast cancer rats. The normal control group rats did not show any sign of tumor incidence. DMBA-induced group rats showed 100% tumor incidence and AX showed decreased tumor incidence of 75% and 46% at a dose level of 5 mg/kg and 50 mg/kg, respectively. AX-SLN-treated group rats demonstrated 9% tumor incidence.

Figure 1(c) shows the tumor volume of different groups of rats. The normal control group rats did not show the tumor volume due to the absence of tumor in the mammary gland tissue. DMBA-induced group rats showed 4.56 cm^3^ tumor volume and AX exhibited 3.88 cm^3^ and 3.05 cm^3^ at a dose of 5 and 50 mg/kg, respectively. AX-SLN showed 0.96 cm^3^.

Figure 1(d) shows the tumor weight of DMBA-induced breast cancer rats. The normal control group rats did not show any sign and symptom of the tumor. DMBA-induced group rats showed an average tumor weight (18.84 g) and AX-SLN significantly (*p* < .001) reduced the tumor weight (2.16 g).

### Effect on the antioxidant parameters

Supplementary Table 5 shows the effect of AX-SLN on the antioxidant parameter of normal and DMBA-induced breast cancer rats. In the current experimental study, we estimated the antioxidant parameter in the serum and different tissues such as mammary gland, liver, and renal. The antioxidant parameters were LPO, SOD, MnSOD, CuZnSOD, CAT, GPx, and GSH. DMBA-induced group rats showed an increase in the level of LPO and a reduced level of SOD, MnSOD, CuZnSOD, CAT, GPx, and GSH in the serum and other tissues such as mammary gland, liver, and renal. AX-SLN significantly (*p* < .001) reduced the LPO and increased the level of SOD, MnSOD, CuZnSOD, CAT, GPx, and GSH in the serum and other tissues such as mammary gland, liver, and renal (Supplementary Table 5).

### Effect on lipid parameters

Supplementary Table 6 demonstrates the effect of AX-SLN on the lipid parameters. The normal control group showed the unaltered lipid profile throughout the experimental study. DMBA-induced breast cancer rats exhibited the increased level of total cholesterol, triglyceride, and high-density lipoprotein, and AX-SLN treatment significantly (*p* < .001) reduced the level of total cholesterol, triglyceride, and high-density lipoprotein almost near to the normal control group rats.

### Effect on glycoprotein

Glycoprotein enzymes such as hexose, sialic acid, and hexosamine were estimated at the end of the experimental study. DMBA-induced group rats showed an increased level of hexose, sialic acid, and hexosamine, and AX-SLN treatment significantly (*p* < .001) reduced the level (Supplementary Table 6).

### Effect on I and II biotransformation enzymes

Supplementary Table 6 exhibits the effect of AX-SLN on phase I and II biotransformation enzymes. DMBA-induced group rats showed an increased level of Cytochrome p450, GST, and decreased level of cytochrome b. AX-SLN significantly (*p* < .001) reduced the level of cytochrome p450, GST, and increased the level of cytochrome b.

### Effect on mitochondrial TCA cycle enzymes

Mitochondrial TCA cycle enzymes such as ICDH, SHD, MDH, and α-KGDH were estimated at the end of the experimental study. DMBA-induced breast cancer rats showed up-regulated levels of ICDH, SHD, MDH, and α-KGDH, and AX-SLN significantly (*p* < .001) down-regulated the level of mitochondrial TCA cycle enzymes such as ICDH, SHD, MDH, and α-KGDH (Supplementary Table 6).

### Effect on carbohydrate metabolizing enzymes

DMBA-induced breast cancer rats showed an increased level of hexokinase, P-glucoisomerase, aldolase, and reduced level of glucose-6-phosphatase and fructose 1-6, biphosphate. AX-SLN significantly (*p* < .001) decreased the level of hexokinase, P-glucoisomerase, and aldolase and enhanced the level of glucose-6-phosphatase and fructose 1-6, biphosphate (Supplementary Table 6).

### Effect on Na+/K+, Ca2+, and Mg2+ activity

DMBA-induced group rats showed the increased activity of Na+/K+, Ca2+, and Mg2+, and AX-SLN significantly (*p* < .001) reduced the activity (Supplementary Table 6).

### Effect on pro-inflammatory cytokines

Supplementary Table 7 shows the effect of AX-SLN on the DMBA-induced breast cancer on the serum, mammary gland, liver, and renal tissue. DMBA-induced rats exhibited an increased level of pro-inflammatory cytokines, and AX-SLN significantly (*p* < .001) reduced the level of pro-inflammatory cytokines in the serum, mammary gland, liver, and renal tissue.

### Effect on hepatic parameters

**Figure 2.**
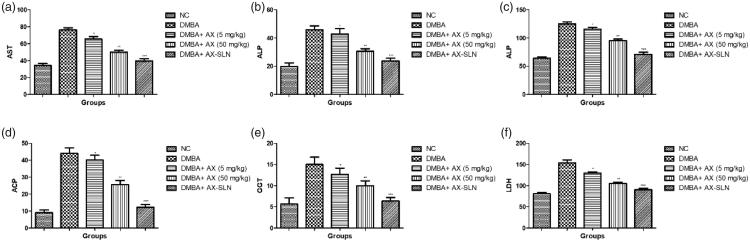
The effect of AX-SLN on the biochemical parameters of DMBA-induced breast cancer rats. (a) AST; (b) ALP, (c) ALT, (d) ACP, (e) GGT, and (f) LDH. Each value shows the mean ± SEM, where the treated group rats were compared with the DMBA-induced group rats. Statistical analysis was done by one-way ANOVA followed by Dunnett’s multiple comparison. *p < .05, **p < .01, and ***p < .001.

Figure 2 exhibits the effect of AX-SLN on the biochemical parameters on the DMBA-induced breast cancer rats. Hepatic parameters such as AST, ALP, ALP, and ACP considerably increased during the DMBA-induced breast cancer, and AX-SLN significantly (*p* < .001) reduced the level of hepatic parameters.

### Effect of AX-SLN on protein expressions of LXR α, LXR β, Maf1, and PTEN

**Figure 3.**
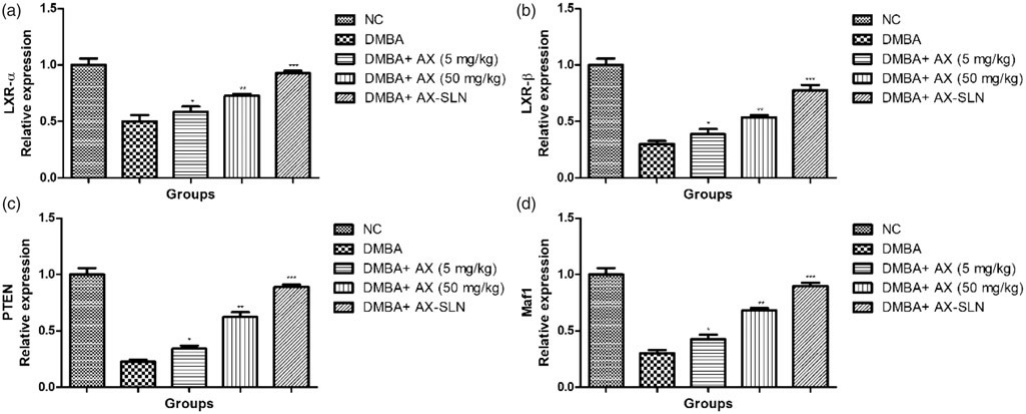
The effect of AX-SLN on the protein expression of LXR (α and β), Maf1 and PTEN. Each value shows the mean ± SEM, where the treated group rats were compared with the DMBA-induced group rats. Statistical analysis was done by one-way ANOVA followed by Dunnett’s multiple comparison. *p < .05, **p < .01, and ***p < .001.

Figure 3 shows the effect of AX-SLN on protein expression of LXR α, LXR β, Maf1, and PTEN on the DMBA-induced breast cancer rats. DMBA-induced rats exhibited the reduced expression of LXR α, LXR β, Maf1, and PTEN, and AX-SLN treatment significantly (*p* < .001) increased the expression almost near to the normal control.

### Effect of AX-SLN on the protein expression of antioxidant marker

**Figure 4.**
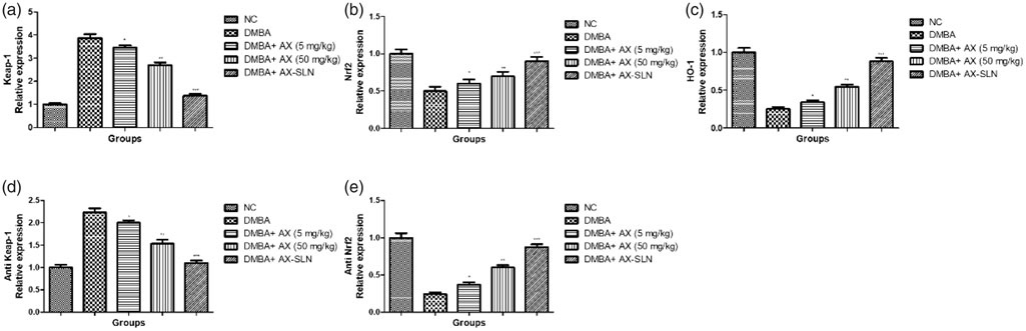
The effect of AX-SLN on the protein expression of antioxidant marker of DMBA-induced breast cancer rats. (a) Keap-1; (b) Nrf2, (c) HO-1, (d) Antikeap-1, and (e) AntiNrf2. Each value shows the mean ± SEM, where the treated group rats were compared with the DMBA-induced group rats. Statistical analysis was done by one-way ANOVA followed by Dunnett’s multiple comparison. *p < .05, **p < .01, and ***p < .001.

During the DMBA-induced breast cancer, the oxidative stress increased and reduced the level of antioxidant. A similar result was observed in our experimental study. DMBA-induced rats demonstrated the increased level of Keap-1, anti Keap-1, and reduced level of HO-1, Nrf2, anti-Nrf2, and AX-SLN treatment significantly (*p* < .001) down-regulated the expression of Keap-1, anti Keap-1 and up-regulated the expression of HO-1, Nrf2, and anti-Nrf2 (Figure 4, blot not shown).

### Effect of AX-SLN on expression of Pi3K, phospho AKT, phospho mTOR, and HMG-CoA reductase

**Figure 5.**
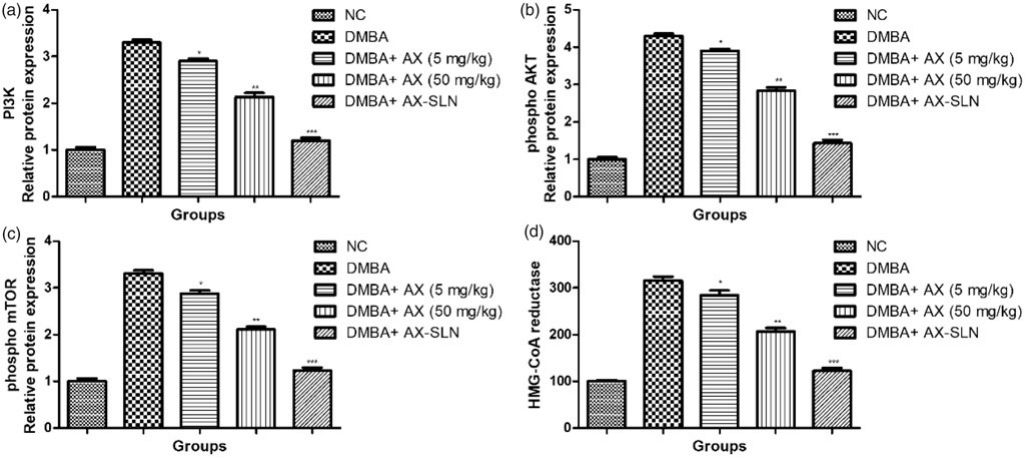
The effect of AX-SLN on the protein expression of Pi3K, phospho AKT, phospho mTOR, and HMG-CoA reductase of DMBA-induced breast cancer rats. (a) Pi3K; (b) phosphor AKT, (c) phosphor mTOR, and (d) HMG-CoA. Each value shows the mean ± SEM, where the treated group rats were compared with the DMBA-induced group rats. Statistical analysis was done by one-way ANOVA followed by Dunnett’s multiple comparison. *p < .05, **p < .01, and ***p < .001.

DMBA-induced breast cancer rats showed increased expression of Pi3K, phospho AKT, phospho mTOR, and HMG-CoA reductase, and AX-SLN significantly (*p* < .001) reduced the expression of Pi3K, phospho AKT, phospho mTOR, and HMG-CoA reductase (Figure 5, blot not shown).

### Effect of A-SLN on the expression of anti HO-1, anti NF-kB, anti p-AKT, and anti p-ERK1/2

**Figure 6.**
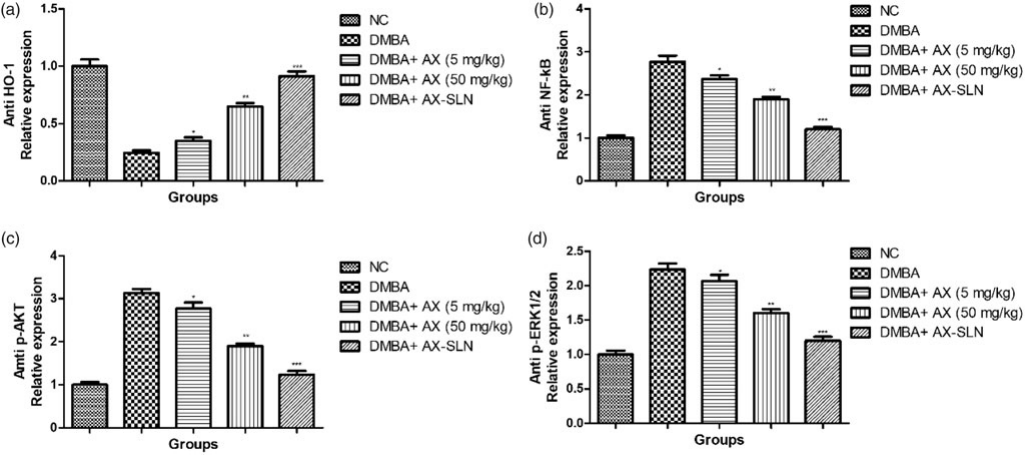
The effect of AX-SLN on the protein expression of anti-HO-1, anti-NF-kB, anti-p-AKT, and anti-p-ERK1/2 of DMBA-induced breast cancer rats. (a) Anti-HO-1; (b) anti-NF-kB, (c) anti-p-AKT, and (d) anti-p-ERK1/2. Each value shows the mean ± SEM, where the treated group rats were compared with the DMBA-induced group rats. Statistical analysis was done by one-way ANOVA followed by Dunnett’s multiple comparison. *p < .05, **p < .01, and ***p < .001.

DMBA-induced group rats exhibited increased expression of anti HO-1 and decreased expression of anti-NF-kB, anti-p-AKT, and anti-p-ERK1/2, and AX-SLN significantly (*p* < .001) reduced the expression of anti-HO-1 and increased the expression of anti-NF-kB, anti-p-AKT, and anti-p-ERK1/2 (Figure 6, blot not shown).

## Discussion

After origination of carcinogenesis, cancer cells divide and propagate in a number of new cells resulting in an increase in the massive tumor burden (Darlington, 1948). Fast-growing neoplastic cells start the alteration of numerous metabolic pathways, involved in the lipogenesis and other energy regulation, to complete the basic requirement for proliferation (Raulet & Guerra, 2009; Vincent et al., 2013). Research suggests that the LXR plays a crucial role in the physiological regulation of carbohydrate metabolism and lipid maintenance, and also suggest that the activation of LXR altered the progression of cancer cell (Xu et al., 2005; Korach-Andre et al., 2011). The researcher suggests that the LXR agonists have been extensively studied to restore the modulation of inflammatory pathway, lipogenic, and metabolic alteration in the proliferation of cancerous condition (Korach-Andre et al., 2011; Korach-André et al., 2011). The researchers target the LXR for the conformational changes that induce the co-activator for corepressor complex exchange and target genes transcription. Researchers also suggest that the LXR activation suppresses the pro-inflammatory cytokines which do not contain the LXR response elements (LXREs), a phenomenon referred as trans repression, and also regulates the intestinal cholesterol absorption via various transporters (Xu et al., 2005; Korach-Andre et al., 2011; Korach-André et al., 2011).

DMBA-induced breast cancer model possesses high human significance which allowed us to scrutinize and explore the underlying mechanisms of numerous phytoconstituents behind their chemoprotective and antioxidant effects (Hilakivi-Clarke et al., 1996). A previous research suggests that the DMBA-induced breast cancer generates numerous reactive intermediates such as OH, O2, and H2O2, and after metabolism they potentiate the oxidative stress primarily via binding to the nucleophilic sites of cellular macromolecules resultant in adenocarcinoma in the mammary gland comparable to the human breast carcinoma (He et al., 2014; Pantavos et al., 2015). The endogenous antioxidant defense system is unable to neutralize the excess generation of ROS and oxidative stress that brings a need for external potential antioxidant. The imbalance of endogenous oxidative status leads to the activation of the anti-oxidative defense system and repairs mechanism mediated by various specific signaling pathways like nuclear factor kappa B (NF-kB) and Kelch-like ECH-associated protein 1 nuclear factor erythroid2-related factor-2 (Keep-1-Nrf2-ARE) pathway (Hayes & McMahon, 2009; Bryan et al., 2013). A previous research suggests that various enzymes and factors have been involved and induced the counteract or paradoxical effects due to the initiation of oxidative reaction (Hayes & McMahon, 2009; Solis et al., 2010; Magesh et al., 2012). A research suggests that the suppression of Nrf2 and enhancement of Keap-1 induce the cellular susceptibility towards ROS, which promotes the inflammatory reaction or immune in the carcinogenesis process (Singh et al., 2006; Hayes & McMahon, 2009; Solis et al., 2010; Linker et al., 2011). A previous research suggests that the overexpression and up-regulation of these inflammatory mediators namely NF-kB, pro-inflammatory cytokines, and immune mediators which then promotes the metastasis and malignancy of diseases (Hur & Gray, 2011; Gañán-Gómez et al., 2013; Davies et al., 2016). Moreover, the alteration of Nfr2 gene directly or indirectly activated the NF-kB-mediated tumorigenesis progression in breast cancer. Due to the effect of NF-kB, Keap-1 on the endogenous antioxidant mechanism may contribute to boost the cytoprotective and chemotherapeutic effect of the anticancer drugs. ROS mediated the activation of inflammatory factor NF-Kb via different intermediate pathways and targeting the ROS mediated commonly used for scrutinized the chemo-protective effect.

Free radical/reactive oxygen species (ROS)/reactive nitrogen species (RNS) play an important role in the expansion of carcinogenesis effect (Afzal et al., 2017; Kumar et al., 2017). Overproduction of free radicals induces the more peroxidation of cellular lipids resultant and enhances the aldehyde such as MDA (an indicator of lipid peroxidation). Excessive generation of MDA in the tissue and serum can be correlated with invasion and proliferation. SOD and CAT play a significant role in the protection of free radical generation. SOD and CAT sequentially modulate the superoxide radical (O_2_) into the hydrogen peroxide (H_2_O_2_), and, ultimately, to water and molecular oxygen, respectively (Anwar et al., 2015; Kumar et al., 2017; Pandey et al., 2018). Researchers suggest that the CuZnSOD and MnSOD (cell constituent of SOD) are found in the mitochondrial matrix and cytosol, respectively. An increased level of superoxide radical decreases the activity of CAT and SOD and boosts the carcinogenesis effect of a toxicant. An increase in the free radical in the serum increases the utilization of SOD and CAT. Enhanced oxidative stress also increased the burden of utilization of endogenous antioxidant due to the formation of a large amount of production of free radicals (Kumar et al., 2018; Pandey et al., 2018). The increased production of free radicals brings critical modification to the different transcription factors such as NF-kB and Nrf-2 that lead to the sequential reaction resultant in the reduction of various cellular antioxidant enzymes. During the current experimental study, we have found a reduction in the SOD and CAT activities in the DMBA control group rats due to carcinogenesis-induced oxidative imbalance and the treatment of astraxanthin increased the activity of SOD and CAT suggesting reduced the oxidative imbalance.

Glutathione peroxidase (GPx), ubiquitous (essential tripeptide) endogenous antioxidant enzyme, is synthesized in the body in a huge amount and takes part in the clearance of cellular peroxidase, carcinogens, and xenobiotics via conjugating toxic substances with GSH as substrates on expenses of H_2_O_2_ (Drevet, 2006; Lubos et al., 2011). During the carcinogenesis effect, the activity of GPx reduced and started the induction of intracellular peroxide deposition in the body that further potentiates the degradation of cross-linking of lipids, polyunsaturated fatty acid, nucleic acid, and proteins. It acts as an imperative intracellular antioxidant and protects the body from the free radical-induced cellular damage and produces substrates for GPx (Bouayed & Bohn, 2010; Varì et al., 2011). DMBA-induced mammary gland group rats showed reduced activity of GPx and GSH which may be due to free radical-mediated up-regulation of and in protecting SH-containing proteins from lipid peroxidase. Astraxanthin significantly (*p* < .001) increased the activity of GSH and GPx, suggesting the antioxidant effect via alteration of free radical generation.

A previous research suggests that HMG-CoAR plays an important role in the progression of breast cancer. It is the rate-limiting enzyme of the mevalonate pathway and also involved in the generation of numerous fundamental end products such as cholesterol and isoprenoids (Harrison et al., 2010; Esquivel-Velázquez et al., 2015). Currently, the researcher focuses on their research targeting the HMG-CoAR inhibition in cancerous cells (Shipitsin et al., 2007). A previous clinical study suggests that the statins (specific inhibitor of HMG-CoAR) have been widely scrutinized for the determination of cancer progression (Shipitsin et al., 2007). During the normal process, the cell regulated the mevalonate pathway and homeostatic feedback response is triggered by statin-induced HMG-CoA reductase and restored the mevalonate pathway. In the tumor cells, they boost the activity of HMG-CoAR via regulation of HMG-CoAR that may induce the de-regulation of mevalonate pathway (Shipitsin et al., 2007; Hartwell et al., 2013). Based on the current fact, in this current experimental study, we have estimated the suppressive effect of astraxanthin on HMG-CoAR and explored the anticancer effect of astraxanthin with the regulation of altered cholesterol metabolism in the mammary gland. In the current study, astraxanthin significantly (*p* < .001) reduced the cholesterol level and suggests the anticancer effect.

During the carcinogenesis effect, PI3K/Akt/mTOR pathway plays a significant role. In the carcinogenesis effect, this regulated various cellular processes such as proliferation, growth, and motility (Miller et al., 2011). AkT phosphorylation plays a crucial role in the process of cell survival. The Akt phosphorylation is reduced when astraxanthin is activated at the level of lipid rafts (Caron et al., 2009). In the current study, the mRNA expression of Akt was reduced as observed in the disease control group and astraxanthin significantly increased the mRNA expression of Akt. PTEN (most common tumor suppressor) is reduced during the cancerous condition. It also regulated lipid and glucose metabolism. Decreased PTEn activity induced via metabolic reprograming of cells facilitates macromolecules synthesis that is required for cell growth. PTEN induces the accumulation of substrate at cellular membrane recruiting Akt and, thereby, activates different downstream signaling pathways supporting cell growth and cell survival (Lim et al., 2003). Maf 1, most important transcription factor, reduced the level of PTEN that inhibited the lipid bio-synthesis, RNA synthesis, and tumor growth. Maf 1 activates the PTEN transcription leading to reduce Akt-mTOR pathway and suppressing the cancer proliferation (Panka et al., 2008). DMBA-induced control group rats exhibited the up-regulation of PI3K, Akt, and mTOR, and down-regulation the Maf-1 and PTEN mRNA expression. Astraxanthin significantly (*p* < .001) down-regulated the PI3K, Akt, and mTOR, and up-regulated the Maf-1 and PTEN mRNA expression.

Nearby lipid, cancer cells circulate the glucose metabolic pathway for the generation of a huge quantity of energy (ATP). Glycolytic pathway plays a crucial role in the generation of ATP and increased the level of Akt. A previous research suggests that the ROS acts as the secondary messenger for activating various signaling mediators like phosphatase and tensin homolog that are deleted on chromosome (PTEN), mitogen-activated protein kinases (MAPKs), and phosphatidylinositol 3 kinase (PI3K), which actively take part in the activation of NF-kB signaling pathway. DMBA-induced breast cancer control group rats exhibited increased expression of p-ERK1/2 and p-AKT, and AX-SLN significantly reduced the expression of p-ERK1/2 and p-AKT. Due to the direct effect of glycolytic pathway on cancer, researchers are targeting the pathway to treat the cancer expansion. Hexokinase, multifunctional protein, performed a crucial role in the transcription regulation and also involved in the apoptosis. During the cancer progression, the expression of HK increases and takes part to increase glycolysis and cell proliferation. The Akt mobilized the cell surface that leads to an increase in the HK activation and glucose uptake that induces the intracellular glucose trapping and phosphorylation. Cancerous tissue expresses all isoenzymes of aldolase but form A is the most protuberant and down-regulation of aldolase activity decreases the cell proliferation of cancerous cells. The suppression of glycolytic enzymes may also reduce glucose consumption. DMBA-induced group rats showed increased activity of AL and HK and astraxanthin significantly (*p* < .001) reduced the activity of AL and HK. On the basis of current finding, we can say that astraxanthin exhibited anti-cancer effect.

Clinical studies suggest that pro-inflammatory cytokines such as TNF-α, IL-1β, and IL-6 play an important role in the expansion of ROS-mediated oxidative stress and inflammatory reactions. Pro-inflammatory cytokines may facilitate the toxic response and deteriorative injury in the tissue via activation of inflammatory mediators (Anwar et al., 2015; Kumar et al., 2017). Moreover, the expansion of inflammatory reaction leads to the deactivation of IKBa inducing the NF-kB activation genes and its subunits to promote the progression of metastatic tumors and malignant. A previous research suggests that inflammatory signaling pathways are involved in the carcinogenesis process via activation of NF-kB transcription factor. During the normal physiological process, IKBa binds to the NF-kB submits and restricts the translocation and activates the NF-kB. During cancer, the ROS and other stress conditions such as COX-2, PGE2, pro-inflammatory cytokines like TNF-α, IL-1β, IL-6, and another NF-kB modulator take part in facilitating the activation of nuclear translocation of NF-kB via numerous mechanisms. A previous research suggests that the RPS plays a crucial role in the activation of NF-kB; after the activation, NF-kB turns numerous specific genes related to the immune response or inflammatory reaction and cell proliferation (Anwar et al., 2015; Afzal et al., 2017; Kumar et al., 2017). Therefore, the mechanism is still unclear to establish the connection between the NF-kB-activated inflammatory changes and various mammary carcinoma motivating toward the metastasis. But a few researchers suggest that the inhibition of inflammatory pathway could be used for the effectiveness of chemoprevention and cancer therapy. In the current experimental study, we have found the increased level of pro-inflammatory cytokines in the DMBA-induced group rats and AX-SLN significantly (*p* < .001) down-regulated the level of the pro-inflammatory cytokine. The higher expression of NF-kB was observed in the normal control group and DMBA-induced breast cancer group rats showed a reduced level of NF-kB. The similar result was observed in the previous research and suggests the expansion of DMBA-induced tumorigenesis. On the contrary, AX-SLN-treated group rats showed reduced inflammatory reactions and impede the nuclear translocation of NF-kB.

Nrf-2 (mediator of Keap1-Nrf2-ARE signaling pathway) plays a significant role in the inflammatory reactions and carcinogenesis-mediated inflammation. During the normal condition, Nrf2 found to be inactivated in the cytoplasm in association with Keap1. During the carcinogenesis effect, the inflammatory reaction and ROS activated the Nrf2 (Solis et al., 2010; Magesh et al., 2012). Activation of Nrf-2 further activated the NF-kB via their directly or indirectly effects. The activation of Nrf2 starts the deposition of a sequence of antioxidative, protein synthesis, and cytoprotective protein like GPx, HO-1, and GST. Various chemotherapeutic agents showed their antioxidant effect via altering the Nfr2 expression (a basic leucine zipper transcription factor that binds to the promoter sequence) (Hayes & McMahon, 2009; Solis et al., 2010; Magesh et al., 2012). Moreover, the activation of Keap1-Nrf2-ARE pathway decreases the intensity of inflammatory reaction and induces the perseverance to present the transformation of acute pathologic conditions into the chronic disease. A previous report suggests that the expression of Keap1 increased and reduced the expression of Nrf2 in the DMBA-induced breast cancer group rats. A similar result was also observed in our experimental study. In the current experimental study, AX-SLN reduced the Keap1 expression and increased the expression of Nrf2, suggesting the anti-inflammatory effect via altering the NF-kB pathway.

## Conclusion

On the basis of the results, we can conclude that astraxanthin significantly (*p* < .001) increased the body weight and reduced the tumor burden. AX-SLN significantly (*p* < .001) increased the body weight and suppresses the tumor in the mammary gland tissue. AX-SLN exhibited altered endogenous antioxidant, pro-inflammatory cytokines, and inflammatory mediators. AX-SLN altered the cancer-induced metabolism and lipogenesis of lipids via re-establishing the level of lipoprotein and lipids. AX-SLN significantly altered the HMG-CoAR. AX-SLN significantly (*p* < .001) up-regulated HO-1 and Nrf-2, and down-regulated the Keap1 expression suggesting the antioxidant effect. Moreover, AX-SLN significantly condenses the AKT-mediated NF-kB expression that contributes to the expansion of mammary gland cancer without altering the p-ERK1/2 levels. Overall, our experimental study suggests the antiproliferative, antioxidant, chemotherapeutic, and anti-inflammatory effects of AX-SLN on DMBA-induced mammary gland tissue in Wistar rats.

## Supplementary Material

Supplemental Material

Supplemental Material

## Figures and Tables

**Table 1.** Effect of AX-SLN on the tumor incidence of DMBA-induced breast cancer rats.

S. no.	Parameters	Groups				
		NC	DMBA	DMBA + AX (5 mg/kg)	DMBA + AX (50 mg/kg)	DMBA + AX-SLN
Pulmonary metastases						
1	Affected rats/total	12/12	11/11	7/12	4/9	2/8
2	Incidence (%)	0	100	58.45	45.49	25
Lymph node metastases						
1	Affected rats/total	12/12	11/11	6/12	3/8	1/8
2	Incidence (%)	0	100	50	37.54	12.5
Adenocarcinoma						
1	Affected rats/total	12/12	11/11	5/11	3/8	1/8
2	Incidence (%)	0	100	45.47	37.54	12.5

**Table 2.** Effect of AX-SLN on the tumor incidence of DMBA-induced breast cancer rats.

S. no	Parameters	Groups				
		NC	DMBA	DMBA + AX (5 mg/kg)	DMBA + AX (50 mg/kg)	DMBA + AX-SLN
1	No of rats with tumors/total rats	0/12	11/11	7/12	4/9	2/8
2	Total Tumor burden (g)	–	96.5	72.5	45.62	10.3

**Table 3.** Effect of AX-SLN on the antioxidant parameters of DMBA-induced breast cancer.

S. no.	Parameters	Groups				
		NC	DMBA	DMBA + AX (5 mg/kg)	DMBA + AX (50 mg/kg)	DMBA + AX-SLN
Serum						
1	LPO	2.5 ± 0.89	3.5 ± 0.98	3.3 ± 0.73*	2.9 ± 0.81***	2.6 ± 0.63***
2	SOD	10 ± 1.89	7 ± 1.04	7.6 ± 1.34**	8.4 ± 1.03***	9.6 ± 0.98***
3	MnSOD	2.1 ± 0.51	1.6 ± 0.34	1.68 ± 0.65*	1.84 ± 0.73***	2.03 ± 0.89***
4	CuZnSOD	8 ± 1.93	5 ± 0.74	5.6 ± 0.45**	6.8 ± 0.43***	7.8 ± 0.34***
5	CAT	60 ± 3.45	38 ± 1.34	45.3 ± 1.04**	51.8 ± 1.38***	59.3 ± 0.93***
6	GPx	9 ± 1.05	4.3 ± 0.85	4.6 ± 0.34**	6.4 ± 0.83***	8.8 ± 0.87***
7	GSH	13 ± 1.32	8.8 ± 1.04	9.3 ± 0.83**	10.7 ± 0.93***	13.9 ± 0.73***
Mammary gland						
1	LPO	3.2 ± 0.53	4.7 ± 1.09	4.4 ± 0.98**	4 ± 0.78***	3.3 ± 0.45***
2	SOD	18.4 ± 1.14	12.3 ± 1.05	13.4 ± 1.11**	15.5 ± 1.03***	17.8 ± 0.94***
3	MnSOD	6.8 ± 0.87	2.3 ± 0.45	2.6 ± 0.34**	4.7 ± 0.45***	6.2 ± 0.67***
4	CuZnSOD	13.2 ± 0.65	9.3 ± 0.56	9.9 ± 0.83**	11.4 ± 0.64***	12.7 ± 0.73***
5	CAT	63 ± 2.34	40.3 ± 3.45	44.5 ± 2.34*	50.3 ± 3.02***	60.4 ± 2.45***
6	GPx	10 ± 1.06	6.3 ± 1.04	6.8 ± 0.83**	7.9 ± 0.64***	9.5 ± 0.89***
7	GSH	13.1 ± 0.98	8.01 ± 0.94	8.45 ± 0.83*	10.3 ± 0.98***	12.8 ± 0.83***
Liver						
1	LPO	1.1 ± 0.23	2.3 ± 0.86	2.1 ± 0.45*	1.7 ± 0.37***	1.2 ± 0.39***
2	SOD	5.3 ± 0.34	2 ± 0.83	2.4 ± 0.89**	3.2 ± 0.83***	4.8 ± 0.84***
3	MnSOD	2.1 ± 0.45	0.5 ± 0.1	0.9 ± 0.2**	1.2 ± 0.1***	2 ± 0.3***
4	CuZnSOD	5.1 ± 0.34	1 ± 0.1	1.5 ± 0.35*	2.8 ± 0.3***	4.9 ± 0.4***
5	CAT	55.4 ± 1.93	42.1 ± 2.04	44.9 ± 1.93**	48.5 ± 2.98***	53.4 ± 2.39***
6	GPx	5.3 ± 0.3	0.4 ± 0.1	1.1 ± 0.1**	2.4 ± 0.3***	4.5 ± 0.39***
7	GSH	10.3 ± 0.5	7.8 ± 0.34	8.3 ± 0.34**	8.9 ± 0.98***	10 ± 0.92***
Renal						
1	LPO	2.5 ± 0.2	3.6 ± 0.45	3.3 ± 0.84*	3 ± 0.74***	2.6 ± 0.84***
2	SOD	7.2 ± 0.12	5.1 ± 0.25	5.4 ± 0.45**	6.2 ± 0.63***	7.1 ± 0.98***
3	MnSOD	2.1 ± 0.10	0.7 ± 0.19	1 ± 0.23**	1.4 ± 0.23***	2.05 ± 0.49***
4	CuZnSOD	5.3 ± 0.14	2.4 ± 0.24	2.8 ± 0.45**	3.9 ± 0.93***	5.1 ± 0.98***
5	CAT	40.3 ± 3.04	28.3 ± 2.12	31.4 ± 2.39*	35.3 ± 2.09***	39.4 ± 2.18***
6	GPx	7.3 ± 0.92	4.8 ± 0.83	5.1 ± 1.24*	5.5 ± 1.45***	7.2 ± 1.23***
7	GSH	13.1 ± 0.56	9.9 ± 0.78	10.4 ± 0.98**	11.1 ± 1.45***	13 ± 2.34***

LPO (mmol/mg of protein); SOD (units/min/mg of proteins); MnSOD (units/min/mg of proteins); CuZnSOD (units/min/mg of proteins); CAT (µmole of H_2_O_2_ consumed/min/mg of protein); GPx = (µmole of glutathione oxidized/min/mg of protein); GSH = (µg/mg of protein). Each value shows the mean ± SEM, where the treated group rats compared with the DMBA-induced group rats. Statistical analysis by one-way ANOVA followed by Dunnett’s multiple comparison. *p < .05, **p < .01, and ***p < .001.

**Table 4.** Effect of astraxanthin on the lipid parameters, glycoprotein, phase I and II biotransformation enzymes, mitochondrial TCA cycle enzymes, and carbohydrate metabolizing enzymes of DMBA-induced breast cancer.

S. no.	Parameters	Groups				
		NC	DMBA	DMBA + AX (5 mg/kg)	DMBA + AX (50 mg/kg)	DMBA + AX-SLN
Lipid profile						
1	Cholesterol	100.3 ± 2.34	80.4 ± 6.54	83.4 ± 4.32*	89.3 ± 4.95**	98.3 ± 5.32***
2	Triglyceride	125.6 ± 5.39	100 ± 3.89	104.5 ± 5.03*	114.3 ± 6.23***	123.5 ± 4.03***
3	High density lipoprotein	40.2 ± 3.23	81.3 ± 4.28	70.3 ± 3.45*	60.3 ± 4.35***	45.6 ± 3.98***
Mitochondrial TCA cycle enzymes						
1	ICDH	650 ± 15.46	389.4 ± 10.34	412.3 ± 8.93*	512.5 ± 8.45**	612.6 ± 9.03***
2	SDH	203.4 ± 5.93	98.3 ± 5.31	111.3 ± 3.98**	134.6 ± 4.03***	183.4 ± 6.03***
3	α-KGDH	113 ± 4.04	50.3 ± 3.45	61.3 ± 4.59**	74.5 ± 3.94***	104.3 ± 498***
4	MDH	400.3 ± 8.93	198.4 ± 6.09	234.5 ± 7.35**	298.3 ± 6.54***	378.4 ± 5.94***
Glycoprotein						
1	Hexose	222.3 ± 5.45	412 ± 6.78	254.5 ± 5.93*	315.4 ± 6.32***	403.2 ± 5.43***
2	Sailic acid	79.4 ± 2.45	113.4 ± 4.98	104.5 ± 4.34*	91.3 ± 3.98**	80.3 ± 3.45***
3	Hexosamine	51.3 ± 2.09	81.3 ± 3.45	74.5 ± 4.12*	60.3 ± 3.45**	53.5 ± 2.85***
Na+/K+, Ca2+, and Mg2+ activity						
1	Na+/K + ATPase	4.5.5 ± 0.3	1.9 ± 0.21	2.1 ± 0.33*	3.2 ± 0.39***	4.3 ± 0.48***
2	Ca2 + ATPase	5.2 ± 0.34	2.4 ± 0.23	2.8 ± 0.34**	3.5 ± 0.54***	5 ± 0.45***
3	Mg2 + ATPase	4.4 ± 0.34	1.3 ± 0.23	1.8 ± 0.18**	2.8 ± 0.28***	4.2 ± 0.41***
Phase I and II biotransformation enzymes						
5	Cytochorme p450	0.7 ± 0.03	0.3 ± 0.01	0.4 ± 0.02*	0.48 ± 0.18**	0.67 ± 0.21***
6	Glutathione S-transferase	3.5 ± 0.02	2 ± 0.01	2.3 ± 0.03*	2.8 ± 0.04**	3.3 ± 0.02***
7	Cytochrome b5	0.8 ± 0.01	0.2 ± 0.01	0.33 ± 0.03*	0.5 ± 0.05**	0.7 ± 0.02***
Carbohydrate metabolizing enzymes						
1	Hexokinase	12.2.±2.34	35.4 ± 3.23	30.1 ± 2.54*	22.3 ± 3.21**	13.4 ± 2.39```***
2	P-Glucoisomerase	25.3 ± 2.93	65.4 ± 3.04	60.2 ± 2.12*	47.6 ± 2.94***	28.3 ± 4.02***
3	Aldolase	22.3 ± 1.92	48.9 ± 3.45	43.3 ± 3.45*	35.3 ± 3.42***	24.5 ± 4.24***
4	Glucose-6-phaspastase	23.4 ± 1.04	14.5 ± 1.98	16.3 ± 2.03*	18.9 ± 2.03**	22.5 ± 3.02***
5	Fructose 1-6, biphosphate	58.4 ± 4.32	23.3 ± 3.94	28.4 ± 1.34*	34.5 ± 3.45**	56.4 ± 3.45***

Each value shows the mean ± SEM, where the treated group rats compared with the DMBA-induced group rats. Statistical analysis by one-way ANOVA followed by Dunnett’s multiple comparison. *p < .05, **p < .01, and ***p < .001.

**Table 5.** Effect of astraxanthin on the pro-inflammatory cytokine parameters of DMBA-induced breast cancer.

S. no.	Parameters	Groups				
		NC	DMBA	DMBA + AX (5 mg/kg)	DMBA + AX (50 mg/kg)	DMBA + AX-SLN
Serum						
1	TNF-α	90.5 ± 3.89	202.3 ± 5.43	180.2 ± 3.09*	154.5 ± 2.93***	101.3 ± 3.98***
2	IL-6	121.3 ± 2.89	354 ± 3.45	304.5 ± 4.23**	214.5 ± 3.98***	135.4 ± 4.03***
3	IL-1β	41.3 ± 3.01	102 ± 2.34	90.3 ± 2.56*	70.4 ± 2.35***	45.6 ± 1.98***
Mammary gland						
1	TNF-α	51.3 ± 1.29	302.3 ± 3.94	275.5 ± 2.34**	202.3 ± 2.83***	70.4 ± 2.91***
2	IL-6	20.3 ± 0.98	80.3 ± 1.12	70.2 ± 1.15**	48.9 ± 1.34***	24.5 ± 2.03***
3	IL-1β	40.5 ± 0.83	150.4 ± 2.38	138.4 ± 1.83**	98.4 ± 1.39***	45.6 ± 2.93***
Liver						
1	TNF-α	1.1 ± 0.23	2.3 ± 0.86	2.1 ± 0.45*	1.7 ± 0.37***	1.2 ± 0.39***
2	IL-6	5.3 ± 0.34	2 ± 0.83	2.4 ± 0.89**	3.2 ± 0.83***	4.8 ± 0.84***
3	IL-1β	2.1 ± 0.45	0.5 ± 0.1	0.9 ± 0.2**	1.2 ± 0.1***	2 ± 0.3***
Renal						
1	TNF-α	2.5 ± 0.2	3.6 ± 0.45	3.3 ± 0.84*	3 ± 0.74***	2.6 ± 0.84***
2	IL-6	7.2 ± 0.12	5.1 ± 0.25	5.4 ± 0.45**	6.2 ± 0.63***	7.1 ± 0.98***
3	IL-1β	2.1 ± 0.10	0.7 ± 0.19	1 ± 0.23**	1.4 ± 0.23***	2.05 ± 0.49***

Each value shows the mean ± SEM, where the treated group rats compared with the DMBA-induced group rats. Statistical analysis by one-way ANOVA followed by Dunnett’s multiple comparison. *p < .05, **p < .01, and ***p < .001.
