# Supplementary figures and images for: Fabrication and characterization of solid lipid nano-formulation of astraxanthin against DMBA-induced breast cancer via Nrf-2-Keap1 and NF-kB and mTOR/Maf-1/PTEN pathway
Source: Drug Deliv. 2019 Sep 26;26(1):975–88. doi: 10.1080/10717544.2019.1667454 (PMC6781204; doi:10.1080/10717544.2019.1667454)

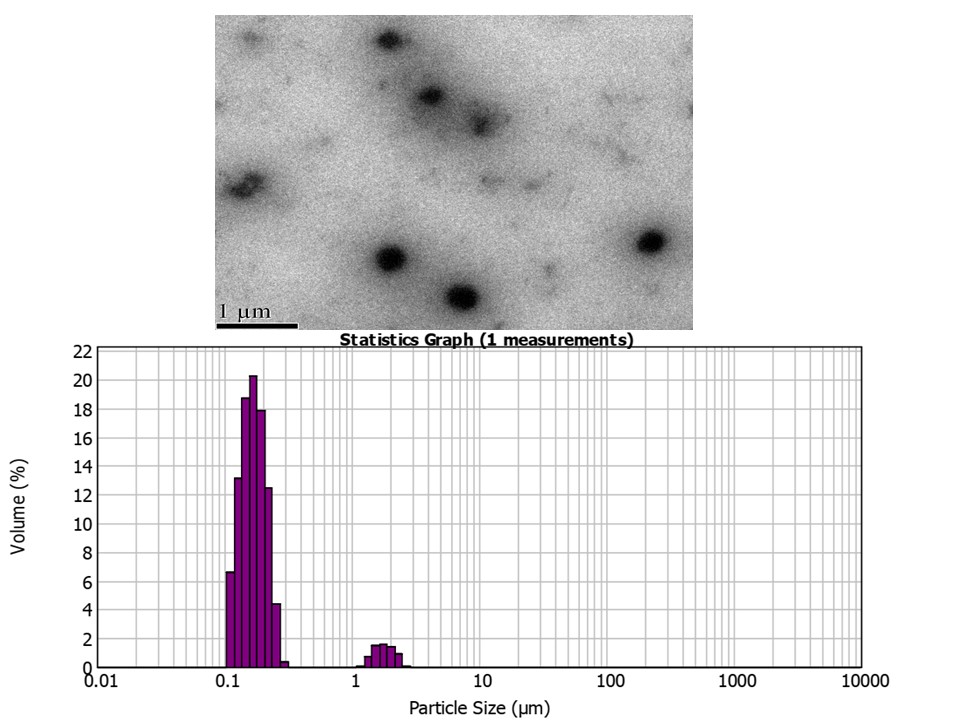


**Supplementary figure 1:** TEM and particle size image of AX-SLN.

Supplement: Supplemental Material [file IDRD_A_1667454_SM3009.docx]
