# Supplementary material for: Fabrication and characterization of solid lipid nano-formulation of astraxanthin against DMBA-induced breast cancer via Nrf-2-Keap1 and NF-kB and mTOR/Maf-1/PTEN pathway
Source: Drug Deliv. 2019 Sep 26;26(1):975–88. doi: 10.1080/10717544.2019.1667454 (PMC6781204; doi:10.1080/10717544.2019.1667454)
Supplement: Supplemental Material [file IDRD_A_1667454_SM3007.docx]

**Supplementary Table 1:** List of primer

| **S. No** | **mRNA** | **Sequence (5′ > 3′)** | |
| --- | --- | --- | --- |
|  |  | **Forward** | **Backward** |
| **1** | **LXRα** | CTCAATGCCTGATGTTTCTCCT | TCCAACCCTATCCCTAAAGCAA |
| **2** | **LXRβ** | ATGTCTTCCCCCACAAGTTCT | GACCACGATGTAGGCAGAGC |
| **3** | **HMG-CoAR** | CTGGAATTATGAGTGCCCCAAA | ACGACTGTACTGAAGACAAAGC |
| **4** | **PTEN** | TGGATTCGACTTAGACTTGACCT | GCGGTGTCATAATGTCTCTCAG |
| **5** | **Maf1** | AGCTACTCGTGTAAGATGGCG | CTGCACTTGTCGCTCAGAG |
| **6** | **PI3K** | AAAGGCCGAGCCCTCTATTAT | GGACAATCTCGACGTAAGAAGC |
| **7** | **Akt** | TGGGTTCAGAAGAGGGGAGAA | AGGGGATAAGGTAAGTCCACATC |
| **8** | **mTOR** | GCTTTGACGCAGGTGCTAAG | TGTCCCCATAACCGGAGTAGG |
| **9** | **GAPDH** | TGGATTTGGACGCATTGGTC | TTTGCACTGGTACGTGTTGAT |

**Supplementary table 2:** *In vitro* skin permeation studies of AX-SLN and control

| Formulation Code | Flux (µg/cm^2^/ hr) | Permeability Co-efficient × 10^-3^(cm/hr) | Enhancement ratio |
| --- | --- | --- | --- |
| AX-SLN | 13.14 | 1.63 | 3.12 |
| Control | 3.12 | 0.25 | 1 |

**Supplementary table 3:** Effect of AX-SLN on the tumor incidence of DMBA induced breast cancer rats.

| **S.No** | **Parameters** | **Groups** | | | | |
| --- | --- | --- | --- | --- | --- | --- |
|  |  | **NC** | **DMBA** | **DMBA+AX (5 mg/kg)** | **DMBA+AX (50 mg/kg)** | **DMBA+AX-SLN** |
| **Pulmonary Metastases** | | | | | | |
| **1** | **Affected Rats/total** | 12/12 | 11/11 | 7/12 | 4/9 | 2/8 |
| **2** | **Incidence (%)** | 0 | 100 | 58.45 | 45.49 | 25 |
| **Lymphnode Metastases** | | | | | | |
| **1** | **Affected Rats/total** | 12/12 | 11/11 | 6/12 | 3/8 | 1/8 |
| **2** | **Incidence (%)** | 0 | 100 | 50 | 37.54 | 12.5 |
| **Adenocarcinoma** | | | | | | |
| **1** | **Affected Rats/total** | 12/12 | 11/11 | 5/11 | 3/8 | 1/8 |
| **2** | **Incidence (%)** | 0 | 100 | 45.47 | 37.54 | 12.5 |

**Supplementary table 4:** effect of AX-SLN on the tumor incidence of DMBA induced breast cancer rats.

| **S.No** | **Parameters** | **Groups** | | | | |
| --- | --- | --- | --- | --- | --- | --- |
|  |  | **NC** | **DMBA** | **DMBA+AX (5 mg/kg)** | **DMBA+AX (50 mg/kg)** | **DMBA+AX-SLN** |
| **1** | **No of rats with tumors/total rats** | 0/12 | 11/11 | 7/12 | 4/9 | 2/8 |
| **2** | **Total Tumor burden (g)** | - | 96.5 | 72.5 | 45.62 | 10.3 |

**Supplementary table 5:** effect of AX-SLN on the antioxidant parameters of DMBA induced breast cancer.

| **S.No** | **Parameters** | **Groups** | | | | |
| --- | --- | --- | --- | --- | --- | --- |
|  |  | **NC** | **DMBA** | **DMBA+AX (5 mg/kg)** | **DMBA+AX (50 mg/kg)** | **DMBA+AX-SLN** |
| **Serum** | | | | | | |
| **1** | **LPO** | 2.5±0.89 | 3.5±0.98 | 3.3±0.73* | 2.9±0.81*** | 2.6±0.63*** |
| **2** | **SOD** | 10±1.89 | 7±1.04 | 7.6±1.34** | 8.4±1.03*** | 9.6±0.98*** |
| **3** | **MnSOD** | 2.1±0.51 | 1.6±0.34 | 1.68±0.65* | 1.84±0.73*** | 2.03±0.89*** |
| **4** | **CuZnSOD** | 8±1.93 | 5±0.74 | 5.6±0.45** | 6.8±0.43*** | 7.8±0.34*** |
| **5** | **CAT** | 60±3.45 | 38±1.34 | 45.3±1.04** | 51.8±1.38*** | 59.3±0.93*** |
| **6** | **GPx** | 9±1.05 | 4.3±0.85 | 4.6±0.34** | 6.4±0.83*** | 8.8±0.87*** |
| **7** | **GSH** | 13±1.32 | 8.8±1.04 | 9.3±0.83** | 10.7±0.93*** | 13.9±0.73*** |
| **Mammary gland** | | | | | | |
| **1** | **LPO** | 3.2±0.53 | 4.7±1.09 | 4.4±0.98** | 4±0.78*** | 3.3±0.45*** |
| **2** | **SOD** | 18.4±1.14 | 12.3±1.05 | 13.4±1.11** | 15.5±1.03*** | 17.8±0.94*** |
| **3** | **MnSOD** | 6.8±0.87 | 2.3±0.45 | 2.6±0.34** | 4.7±0.45*** | 6.2±0.67*** |
| **4** | **CuZnSOD** | 13.2±0.65 | 9.3±0.56 | 9.9±0.83** | 11.4±0.64*** | 12.7±0.73*** |
| **5** | **CAT** | 63±2.34 | 40.3±3.45 | 44.5±2.34* | 50.3±3.02*** | 60.4±2.45*** |
| **6** | **GPx** | 10±1.06 | 6.3±1.04 | 6.8±0.83** | 7.9±0.64*** | 9.5±0.89*** |
| **7** | **GSH** | 13.1±0.98 | 8.01±0.94 | 8.45±0.83* | 10.3±0.98*** | 12.8±0.83*** |
| **Liver** | | | | | | |
| **1** | **LPO** | 1.1±0.23 | 2.3±0.86 | 2.1±0.45* | 1.7±0.37*** | 1.2±0.39*** |
| **2** | **SOD** | 5.3±0.34 | 2±0.83 | 2.4±0.89** | 3.2±0.83*** | 4.8±0.84*** |
| **3** | **MnSOD** | 2.1±0.45 | 0.5±0.1 | 0.9±0.2** | 1.2±0.1*** | 2±0.3*** |
| **4** | **CuZnSOD** | 5.1±0.34 | 1±0.1 | 1.5±0.35* | 2.8±0.3*** | 4.9±0.4*** |
| **5** | **CAT** | 55.4±1.93 | 42.1±2.04 | 44.9±1.93** | 48.5±2.98*** | 53.4±2.39*** |
| **6** | **GPx** | 5.3±0.3 | 0.4±0.1 | 1.1±0.1** | 2.4±0.3*** | 4.5±0.39*** |
| **7** | **GSH** | 10.3±0.5 | 7.8±0.34 | 8.3±0.34** | 8.9±0.98*** | 10±0.92*** |
| Renal | | | | | | |
| **1** | **LPO** | 2.5±0.2 | 3.6±0.45 | 3.3±0.84* | 3±0.74*** | 2.6±0.84*** |
| **2** | **SOD** | 7.2±0.12 | 5.1±0.25 | 5.4±0.45** | 6.2±0.63*** | 7.1±0.98*** |
| **3** | **MnSOD** | 2.1±0.10 | 0.7±0.19 | 1±0.23** | 1.4±0.23*** | 2.05±0.49*** |
| **4** | **CuZnSOD** | 5.3±0.14 | 2.4±0.24 | 2.8±0.45** | 3.9±0.93*** | 5.1±0.98*** |
| **5** | **CAT** | 40.3±3.04 | 28.3±2.12 | 31.4±2.39* | 35.3±2.09*** | 39.4±2.18*** |
| **6** | **GPx** | 7.3±0.92 | 4.8±0.83 | 5.1±1.24* | 5.5±1.45*** | 7.2±1.23*** |
| **7** | **GSH** | 13.1±0.56 | 9.9±0.78 | 10.4±0.98** | 11.1±1.45*** | 13±2.34*** |

LPO (mmol/mg of protein); SOD (units/min/mg of proteins); MnSOD (units/min/mg of proteins); CuZnSOD (units/min/mg of proteins); CAT (µmole of H_2_O_2_ consumed/min/mg of protein); GPx=(µmole of glutathione oxidized/min/mg of protein); GSH=(µg/mg of protein). Each value shows the mean ± SEM; where treated group rats compared with the DMBA induced group rats. Statistical analysis by one-way ANOVA followed by Dunnett’s multiple comparison. *p < 0.05, **p < 0.01 and ***p < 0.001.

**Supplementary table 6:** effect of astraxanthin on the lipid parameters, glycoprotein, phase I and II biotransformation enzymes, mitochondrial TCA cycle enzymes and carbohydrate metabolizing enzymes of DMBA induced breast cancer.

| **S.No** | **Parameters** | **Groups** | | | | |
| --- | --- | --- | --- | --- | --- | --- |
|  |  | **NC** | **DMBA** | **DMBA+AX (5 mg/kg)** | **DMBA+AX (50 mg/kg)** | **DMBA+AX-SLN** |
| **Lipid profile** | | | | | | |
| **1** | **Cholesterol** | 100.3±2.34 | 80.4±6.54 | 83.4±4.32* | 89.3±4.95** | 98.3±5.32*** |
| **2** | **Triglyceride** | 125.6±5.39 | 100±3.89 | 104.5±5.03* | 114.3±6.23*** | 123.5±4.03*** |
| **3** | **High density lipoprotein** | 40.2±3.23 | 81.3±4.28 | 70.3±3.45* | 60.3±4.35*** | 45.6±3.98*** |
| **Mitochondrial TCA cycle enzymes** | | | | | | |
| **1** | **ICDH** | 650±15.46 | 389.4±10.34 | 412.3±8.93* | 512.5±8.45** | 612.6±9.03*** |
| **2** | **SDH** | 203.4±5.93 | 98.3±5.31 | 111.3±3.98** | 134.6±4.03*** | 183.4±6.03*** |
| **3** | **α-KGDH** | 113±4.04 | 50.3±3.45 | 61.3±4.59** | 74.5±3.94*** | 104.3±498*** |
| **4** | **MDH** | 400.3±8.93 | 198.4±6.09 | 234.5±7.35** | 298.3±6.54*** | 378.4±5.94*** |
| **Glycoprotein** | | | | | | |
| **1** | **Hexose** | 222.3±5.45 | 412±6.78 | 254.5±5.93* | 315.4±6.32*** | 403.2±5.43*** |
| **2** | **Sailic acid** | 79.4±2.45 | 113.4±4.98 | 104.5±4.34* | 91.3±3.98** | 80.3±3,45*** |
| **3** | **Hexosamine** | 51.3±2.09 | 81.3±3.45 | 74.5±4.12* | 60.3±3.45** | 53.5±2.85*** |
| Na+/K+, Ca2+, and Mg2+ activity | | | | | | |
| **1** | Na+/K+ATPase | 4.5.5±0.3 | 1.9±0.21 | 2.1±0.33* | 3.2±0.39*** | 4.3±0.48*** |
| **2** | Ca2+ATPase | 5.2±0.34 | 2.4±0.23 | 2.8±0.34** | 3.5±0.54*** | 5±0.45*** |
| **3** | Mg2+ATPase | 4.4±0.34 | 1.3±0.23 | 1.8±0.18** | 2.8±0.28*** | 4.2±0.41*** |
| **Phase I and II biotransformation enzymes** | | | | | | |
| **5** | **Cytochorme p450** | 0.7±0.03 | 0.3±0.01 | 0.4±0.02* | 0.48±0.18** | 0.67±0.21*** |
| **6** | **Glutathione S-transferase** | 3.5±0.02 | 2±0.01 | 2.3±0.03* | 2.8±0.04** | 3.3±0.02*** |
| **7** | **Cytochrome b5** | 0.8±0.01 | 0.2±0.01 | 0.33±0.03* | 0.5±0.05** | 0.7±0.02*** |
| **Carbohydrate metabolizing enzymes** | | | | | | |
| **1** | **Hexokinase** | 12.2.±2.34 | 35.4±3.23 | 30.1±2.54* | 22.3±3.21** | 13.4±2.39```*** |
| **2** | **P-Glucoisomerase** | 25.3±2.93 | 65.4±3.04 | 60.2±2.12* | 47.6±2.94*** | 28.3±4.02*** |
| **3** | **Aldolase** | 22.3±1.92 | 48.9±3.45 | 43.3±3.45* | 35.3±3.42*** | 24.5±4.24*** |
| **4** | **Glucose-6-phaspastase** | 23.4±1.04 | 14.5±1.98 | 16.3±2.03* | 18.9±2.03** | 22.5±3.02*** |
| **5** | **Fructose 1-6, biphosphate** | 58.4±4.32 | 23.3±3.94 | 28.4±1.34* | 34.5±3.45** | 56.4±3.45*** |

Each value shows the mean ± SEM; where treated group rats compared with the DMBA induced group rats. Statistical analysis by one-way ANOVA followed by Dunnett’s multiple comparison. *p < 0.05, **p < 0.01 and ***p < 0.001.

**Supplementary table 7:** effect of astraxanthin on the pro-inflammatory cytokine parameters of DMBA induced breast cancer.

| **S.No** | **Parameters** | **Groups** | | | | |
| --- | --- | --- | --- | --- | --- | --- |
|  |  | **NC** | **DMBA** | **DMBA+AX (5 mg/kg)** | **DMBA+AX (50 mg/kg)** | **DMBA+AX-SLN** |
| **Serum** | | | | | | |
| **1** | **TNF-α** | 90.5±3.89 | 202.3±5.43 | 180.2±3.09* | 154.5±2.93*** | 101.3±3.98*** |
| **2** | **IL-6** | 121.3±2.89 | 354±3.45 | 304.5±4.23** | 214.5±3.98*** | 135.4±4.03*** |
| **3** | **IL-1β** | 41.3±3.01 | 102±2.34 | 90.3±2.56* | 70.4±2.35*** | 45.6±1.98*** |
| **Mammary gland** | | | | | | |
| **1** | **TNF-α** | 51.3±1.29 | 302.3±3.94 | 275.5±2.34** | 202.3±2.83*** | 70.4±2.91*** |
| **2** | **IL-6** | 20.3±0.98 | 80.3±1.12 | 70.2±1.15** | 48.9±1.34*** | 24.5±2.03*** |
| **3** | **IL-1β** | 40.5±0.83 | 150.4±2.38 | 138.4±1.83** | 98.4±1.39*** | 45.6±2.93*** |
| **Liver** | | | | | | |
| **1** | **TNF-α** | 1.1±0.23 | 2.3±0.86 | 2.1±0.45* | 1.7±0.37*** | 1.2±0.39*** |
| **2** | **IL-6** | 5.3±0.34 | 2±0.83 | 2.4±0.89** | 3.2±0.83*** | 4.8±0.84*** |
| **3** | **IL-1β** | 2.1±0.45 | 0.5±0.1 | 0.9±0.2** | 1.2±0.1*** | 2±0.3*** |
| **Renal** | | | | | | |
| **1** | **TNF-α** | 2.5±0.2 | 3.6±0.45 | 3.3±0.84* | 3±0.74*** | 2.6±0.84*** |
| **2** | **IL-6** | 7.2±0.12 | 5.1±0.25 | 5.4±0.45** | 6.2±0.63*** | 7.1±0.98*** |
| **3** | **IL-1β** | 2.1±0.10 | 0.7±0.19 | 1±0.23** | 1.4±0.23*** | 2.05±0.49*** |

Each value shows the mean ± SEM; where treated group rats compared with the DMBA induced group rats. Statistical analysis by one-way ANOVA followed by Dunnett’s multiple comparison. *p < 0.05, **p < 0.01 and ***p < 0.001.
